# Comparison of therapeutic effects of endoscopic assisted different surgical approaches in hypertensive intracerebral hemorrhage: A retrospective cohort study

**DOI:** 10.1097/MD.0000000000037211

**Published:** 2024-02-09

**Authors:** Song Wang, Fei Su, Xiguang Zhou, Long Liu, Ruishan Zhang, Zhensheng Xue

**Affiliations:** a Department of Neurosurgery, Xingtai Third Hospital, Xingtai City, China.

**Keywords:** endoscopic assisted, frontal lobe approach, hypertensive intracerebral hemorrhage, temporal lobe approach

## Abstract

We aimed to explore the therapeutic effects of endoscopically assisted surgical approaches for HICH. In this retrospective cohort study, we retrospectively analyzed the treatment status of 118 patients with HICH who underwent surgery for hematoma removal. Among them, 61 patients underwent endoscopically assisted hematoma removal surgery through the frontal lobe approach (frontal lobe group); 57 patients underwent endoscopic hematoma assisted via the temporal lobe approach (temporal lobe group). Treatment effects, cerebral hemodynamic status before and after treatment, postoperative prognosis at one month, and incidence of complications were compared between the 2 groups. We found that the total effective treatment rate in the frontal lobe group was higher than that in the temporal lobe group (*P* < .05). After surgery, the R during the contraction period of the common cerebral artery in both groups decreased compared to that before surgery, and the frontal lobe group was significantly lower than the temporal lobe group; the V and Q were higher than those before surgery, and the frontal lobe group was significantly higher than the temporal lobe group (*P* < .05). The prognosis of the frontal lobe group was better than that of the temporal lobe group (*P* < .05). Compared to the endoscopic-assisted temporal approach, the endoscopic-assisted frontal lobe approach for the treatment of HICH can improve cerebral hemodynamic status, enhance treatment efficacy, and improve prognosis.

## 1. Introduction

Hypertensive intracerebral hemorrhage (HICH) is a multiple cerebrovascular disease and the most severe non-traumatic bleeding type among acute cerebrovascular diseases.^[1]^ HICH has the characteristics by rapid onset, rapid progression, and severe conditions.^[1,2]^ The onset of HICH is caused by a sharp increase in blood pressure, which leads to the rupture of cerebral arterioles and intracerebral hemorrhage, posing a great threat to the patient’s life and health.^[3]^ After the onset of HICH, the hematoma rapidly expands and damages brain tissue and local nerve cells, leading to brain dysfunction.^[3,4]^ And the expansion of hematoma can compress the surrounding brain tissue, affect vascular circulation, cause hypoxia and ischemia of the brain tissue, and lead to cerebral edema.^[5]^ Brain edema and space-occupying effects can further increase blood pressure, create a vicious cycle, and affect the disease prognosis.^[4–6]^ Therefore, early elimination of the hematoma-occupying effect and elimination of secondary lesions of hematoma are of great significance.

Surgery is an important treatment option for HICH. Open surgery, owing to its large trauma and high incidence of complications, is not conducive to postoperative recovery and gradually fails to meet clinical needs.^[7]^ Minimally invasive puncture is also commonly used in HICH, but it can easily lead to incomplete drainage of the hematoma and a poor prognosis.^[8]^ With the advancement of medical technology and the popularization of minimally invasive concepts. Endoscopic assisted surgery has been widely recognized for its advantages such as minimal trauma, high safety, and fast postoperative recovery.^[9–11]^ However, different approaches have different therapeutic effects and there are no reports indicating which approach has a better prognosis for patients.^[12,13]^ In recent years, our hospital has adopted endoscopic-assisted suprafrontal sulcus and temporal lobe hematoma removal surgery for the treatment of HICH. This study aimed to explore the therapeutic effects and short-term prognosis of 2 different approaches for patients. This provides a reference for selecting more suitable treatment methods in later stages.

## 2. Materials and methods

A total of 118 patients with HICH who underwent hematoma removal surgery at our hospital between February 2021 and May 2023 were retrospectively selected. Among them, 61 patients underwent endoscopic assisted hematoma removal surgery through the frontal lobe approach and were assigned to the frontal lobe group; 57 patients underwent endoscopic thrombectomy via the temporal lobe approach and were assigned to the temporal lobe group.

All procedures performed in the study involving human participants were in accordance with the ethical standards of the institutional and/or national research committee(s) and the Helsinki Declaration (as revised in 2013). The requirement for informed consent was waived by the ethics committee due to the observational and retrospective nature of the study.

### 2.1. Inclusion criteria

- Patients met the HICH diagnostic criteria^[1]^;
- Patients diagnosed through head CT and other examinations;
- Patients with complete clinical data.

### 2.2. Exclusion criteria

- Patients with coagulation dysfunction;
- Patients with hematoma caused by brainstem hemorrhage and intracranial vascular malformations;
- The interval from onset to admission was ≥ 24 hours;
- Patients with significant displacement of midline structure requiring craniotomy for decompressive craniectomy;
- Patients had long term use of anticoagulant drugs;
- Patients with cranial tumors;
- Patients with preoperative brain herniation.

### 2.3. Endoscopic assisted frontal lobe approach

General anesthesia was induced using the German STORZ neuroendoscopic system. A longitudinal incision 1 cm was made before the coronal suture and approximately 3 cm beside the midline, or a small skin flap incision in a “U” shape. Then a small hole with a diameter of about 8 mm was made in the skull and was enlarged into a bone window with a diameter of 3 cm using a milling cutter. The dura mater was cut into a “U” shape to expose the superior frontal sulcus between the superior and middle frontal gyri. Electrocoagulation treatment of the frontal sulcus was performed with a brain puncture needle punctured the hematoma center, the hematoma cavity was cleared, the hematoma was partial extracted, and the intracranial pressure decreased. The brain puncture needle was removed, a soft-tissue expansion tube with an inner core was inserted, and the tube core was withdrawn. A soft tissue expansion tube was inserted into the neuroendoscope to clearly reach the hematoma cavity; the direction and depth of the soft tissue expansion cannula were fixed or adjusted through an assistant or snake-shaped traction arm. With the assistance of the monitoring system, a suction device was used to remove the hematoma in blocks, there was no need to completely remove the hematoma, a small amount of attached hematoma could be retained, and 80% to 90% could be cleared. During treatment, attention should be paid to controlling the direction and force of the suction device to avoid damaging the brain tissue and causing bleeding. If there is active bleeding, extended bipolar electrocoagulation can be used for hemostasis, or weak suction can be maintained through a suction device to suction the bleeding point. Hemostasis can be achieved through a unipolar electrocoagulation point contact suction device. After removal of the hematoma, the hematoma cavity was cleaned with physiological saline. Residual blood from the hematoma cavity was removed as much as possible. Drainage tube does not need to be set aside. The soft tissue expansion tube was removed, the dura mater was sutured, the bone flap for treatment was returned, fixed with a titanium connector, and the scalp was sutured layer by layer.

### 2.4. Endoscopic assisted temporal lobe approach

The patient lied flat and underwent general anesthesia. The head was tilted to one side, an incision approximately 2 cm above the external auditory canal was made to form a small bone window, the posterior temporal gyrus of the dura mater was cut open into the hematoma cavity, and the hematoma was cleared using neuroendoscopy. The relevant procedures were the same as those used for the temporal lobe approach.

Observation data. Therapeutic effects. When the hematoma clearance rate is greater than 80% and the National Institutes of Health Stroke Scale (NIHSS) improvement is ≥ 90%, it has a significant effect; when the hematoma clearance rate is 60% to 80%, NIHSS improvement of 60% to 89% is effective; when the hematoma clearance rate is less than 60% and NIHSS improvement is less than 60%, it is considered ineffective; and significant and effective results are included in the total effective rate. Cerebral hemodynamic status. Peripheral resistance I, mean blood flow velocity (V), and mean blood flow volume (Q) during the contraction phase of the common cerebral artery were measured using a LUTI-T0212 transcranial Doppler detector (DWI, Germany). Prognosis 1 month after surgery. Glasgow Outcome Scale assessment was used: if death is Grade I, if it is a vegetative state, it is classified as Grade II; if the consciousness is clear, severely disabled, and requires care from others in daily life, it is classified as Grade III; if it is a mild disability and can live independently, it is classified as Grade IV; if the recovery is good, it is rated as Grade V; Grade IV and Grade V are included in the good prognosis rate. Occurrence of complications. These include cerebral infarction, intracranial infection, and rebleeding.

### 2.5. Statistical methods

All data analyses were conducted using the SPSS software (version 26.0; IBM Corp., Armonk, NY). The Shapiro–Wilk test was used to evaluate the normality of the data. The data with normal distribution are represented by mean ± standard deviation. Independent sample *t* test was used for inter-group comparison, paired *t* test was used for intragroup comparisons before and after comparison, and the chi-square test was used to represent the number of use cases. When *P* < .05, the differences were considered statistically significant.

## 3. Results

This study included 118 patients, including 62 males and 56 females, aged 48 to 77 years, with an average of 62.52 ± 6.91 years old. There were 61 and 57 cases in the frontal and temporal lobe groups, respectively. There was no significant difference in baseline data between the 2 groups of patients (*P* > .05) (Table 1). The total effective treatment rate in the frontal lobe group (93.44%) was higher than that in the temporal lobe group (78.95%) (*P* < .05) (Table 2). Before surgery, there was no significant difference in hemodynamic indicators between the 2 groups (*P* > .05); After surgery, the R of both groups decreased compared to before, and the frontal lobe group was significantly lower than the temporal lobe group; However, V and Q increased compared to preoperative levels, and the frontal lobe group was significantly higher than the temporal lobe group (*P* < .05) (Table 3). The excellent prognosis rate in the frontal lobe group (72.13%) was higher than that in the temporal lobe group (45.62%) (*P* < .05) (Table 4). The incidence of complications in the frontal lobe group (3.28%) was lower than that in the temporal lobe group (7.02%); however, the difference was not significant (*P* > .05) (Table 5).

**Table 1:** Comparison of baseline data between 2 groups.

Group	Gender (Male/female)	Age (yr)	Blood loss (mL)	Bleeding site	
				Basal ganglia region	Lobe of brain
Frontal lobe group (n = 61)	35/26	63.07 ± 6.64	38.39 ± 5.01	38 (62.30)	23 (37.70)
Temporal lobe group (n = 57)	27/30	61.93 ± 7.20	39.21 ± 5.78	31 (54.39)	26 (45.61)
*χ^2^/t*	1.184	0.892	−0.822	0.759	
*P*	.277	.375	.413	.384	

**Table 2:** Comparison of treatment effects between 2 groups.

Group	n	Significant effect	Effective	Invalid	Total effective rate
Frontal lobe group	61	31 (50.82)	26 (42.62)	4 (6.56)	57 (93.44)
Temporal lobe group	57	20 (35.09)	25 (43.86)	12 (21.05)	45 (78.95)
*χ^2^*					6.264
*P*					.044

**Table 3:** Comparison of cerebral hemodynamic status between 2 groups.

Time	Group	n	R (Pa·s/mL)	V (mL/min)	Q (cm/s)
Before surgery	Frontal lobe group	61	1.85 ± 0.29	915.8 ± 102.5	11.10 ± 2.13
	Temporal lobe group	57	1.79 ± 0.31	908.3 ± 108.1	11.75 ± 2.21
	*t*		1.150	0.389	−1.641
	*P*		.253	.698	.103
After surgery	Frontal lobe group	61	1.40 ± 0.23*	1137 ± 119*	19.15 ± 2.18*
	Temporal lobe group	57	1.57 ± 0.28*	1090 ± 112*	17.23 ± 2.53*
	*t*		−3.686	2.184	4.423
	*P*		<.001	.031	<.001

Compared with before treatment in the same group,

* *P* < .05.

**Table 4:** Comparison of prognosis between 2 groups.

Group	n	Grade I	Grade II	Grade III	Grade IV	Grade V	Excellent prognosis rate
Frontal lobe group	61	0 (0.00)	3 (5.08)	14 (27.12)	39 (59.32)	5 (8.47)	44 (72.13)
Temporal lobe group	57	1 (1.69)	8 (15.25)	22 (35.59)	24 (44.07)	2 (3.39)	26 (45.62)
*χ^2^*							9.783
*P*							.044

**Table 5:** Comparison of complications between 2 groups.

Group	n	Cerebral infarction	Intracranial infection	Rebleeding	Incidence of complications
Frontal lobe group	61	1 (1.69)	0 (0.00)	1 (1.69)	2 (3.28)
Temporal lobe group	57	2 (3.39)	1 (1.69)	1 (1.69)	4 (7.02)
*χ^2^*					0.853
*P*					.356

## 4. Discussion

The results of this study indicate that the use of an endoscopic-assisted frontal lobe approach for the treatment of HICH has high practical value, can improve the treatment effect of the disease, and can reduce the occurrence of complications. Cui et al^[14]^ also confirmed that compared to the temporal approach surgery, the frontal approach surgery for basal ganglia cerebral hemorrhage can more effectively improve the patient’s motor function and ensure a good outcome of the disease. It also points out that the relevant endoscopic-assisted frontal approach surgeries are deep along the long axis of the hematoma, which is beneficial for avoiding or reducing further damage to the relevant white matter fiber bundles.^[14]^ Therefore, it can ensure postoperative functional rehabilitation and avoid affecting the good outcome of the disease. Cao et al^[15]^ found that referring to the morphological characteristics of the lateral ventricle, the frontal approach enters the ventricle from the frontal horn, and by using endoscopy to adjust the angle within a small range, the frontal angle and body of the ventricle can be explored, effectively clearing the accumulation of blood in the ventricles and clearing the accumulation of blood in the contralateral ventricles through a transparent septum. Meanwhile, blood accumulation in the third ventricle can also be cleared through the interventricular foramen. If necessary, a third ventricular floor ostomy can be performed to reduce the risk of hydrocephalus and other complications. Sun et al^[16]^ pointed out that although traditional open surgery can achieve certain results, trauma is significant and not conducive to postoperative recovery, while endoscopic surgery can provide a clear surgical field and reduce surgical trauma. Endoscopic assisted soft tissue channels can adjust direction in a timely manner, resulting in clearer imaging. Moreover, it is beneficial for postoperative hemostasis, reducing damage to brain tissue, and the operation channel of neuroendoscopy is relatively small, which can not only avoid brain tissue damage to the greatest extent, but also protect with the help of soft tissue channels, avoiding iatrogenic injuries and promoting postoperative recovery. At the same time, in the endoscopic-assisted frontal lobe approach, a cortical fistula can be fixed by selecting the suprafrontal sulcus, without the need to adjust the drilling position with the help of the hematoma location, making it convenient for the surgeon to operate. This surgical approach can effectively avoid important functional areas of the frontal cortex and basal ganglia, protect nerve function, prevent compression of the lateral fissure blood vessels, and prevent venous return.^[17]^ Zheng et al^[18]^ also pointed out that the distance to reach the hematoma through temporal surgery is relatively short; however, the surgical path is perpendicular to the long axis of most basal ganglia hematomas, so the working channel needs to swing significantly during surgery, which can increase secondary damage and result in poor clearance of narrow and elongated hematomas. Although the surgical pathway of the frontal approach is relatively long, it is basically parallel to the long axis of the hematoma, which can solve the above shortcomings; however, problems such as decreased puncture accuracy and poor lighting effects caused by a longer surgical path can be solved through endoscopy. In addition, the incidence of complications in the frontal lobe group was slightly lower than that in the temporal lobe group; however, the difference was not significant. The reason for this may be that both groups underwent minimally invasive surgery, which resulted in a lower incidence of complications.^[17,18]^

In addition, the hemodynamic state can reflect the local blood supply in the brains of patients with HICH. Abnormal perfusion of brain tissue blood flow can cause neuronal damage.^[19]^ The results of this study indicate that the endoscopic-assisted frontal lobe approach is superior to the temporal approach in improving cerebral hemodynamic status in patients with HICH. The main reason is that during the implementation of the temporal lobe approach, the placement of a transparent lens sheath can compress the contralateral fissure blood vessels to a certain extent, affecting the venous return of the lateral fissure blood vessels, which is not conducive to the improvement of local blood circulation disorders. The frontal lobe approach can improve the clearance rate of hematoma and improve cerebral blood circulation.^[19,20]^ This study also found that the frontal lobe group had a higher rate of good prognosis than the temporal lobe group did. This indicates that the endoscopy-assisted frontal lobe approach can effectively promote good outcomes in patients with HICH. Main reason: When using the temporal approach to remove an intracerebral hematoma, it is easy to limit the direct viewing angle because of the consistency between the line of sight and the length of the hematoma, thereby reducing the hematoma clearance rate.^[20,21]^ Even by adjusting the direction of neuroendoscopy and using angled neuroendoscopy to increase the visual range, the location of the hematoma can increase brain tissue damage, which is not conducive to disease progression, and the temporal approach is prone to transverse damage to the conduction tract. The line of sight and length of the hematoma in the frontal lobe approach are basically consistent and roughly parallel to the conduction tract, which provides better protection for the conduction tract than the temporal approach, improves neurological function, and ensures the effectiveness of disease prognosis.^[20–22]^

### 4.1. Limitations

First, this was a single-center retrospective study. Incomplete medical records and bias in recalling medical history increase the complexity of the research and may lead to selection bias. Second, neither group was randomly assigned, and baseline information may have been imbalanced and biased, which is also one of the shortcomings of our retrospective study. Third, analyzing only the prognosis 1 month after surgery requires a longer follow-up time to verify the results. Finally, the impact of the 2 methods on the long-term functional recovery of patients was not analyzed. We will continue to conduct high-quality research in the future to verify this conclusion.

## 5. Conclusion

Compared with the endoscopic-assisted temporal approach, the endoscopic-assisted frontal lobe approach for the treatment of HICH can improve cerebral hemodynamic status, enhance treatment efficacy, and improve prognosis.

## Author contributions

**Conceptualization:** Song Wang.

**Data curation:** Fei Su, Xiguang Zhou.

**Investigation:** Fei Su, Long Liu.

**Software:** Ruishan Zhang.

**Writing – original draft:** Song Wang.

**Writing – review & editing:** Zhensheng Xue.
